# Muscle spindle reinnervation using transplanted embryonic dorsal root ganglion cells after peripheral nerve transection in rats

**DOI:** 10.1111/cpr.12660

**Published:** 2019-07-02

**Authors:** Kenichi Asano, Tomonori Nakano, Katsuhiro Tokutake, Hisao Ishii, Takanobu Nishizuka, Katsuyuki Iwatsuki, Tetsuro Onishi, Shigeru Kurimoto, Michiro Yamamoto, Masahiro Tatebe, Hitoshi Hirata

**Affiliations:** ^1^ Department of Hand Surgery Nagoya University Graduate School of Medicine Nagoya Japan

## Abstract

**Objectives:**

Muscle spindles are proprioceptive receptors in the skeletal muscle. Peripheral nerve injury results in a decreased number of muscle spindles and their morphologic deterioration. However, the muscle spindles recover when skeletal muscles are reinnervated with surgical procedures, such as nerve suture or nerve transfer. Morphological changes in muscle spindles by cell transplantation procedure have not been reported so far. Therefore, we hypothesized that transplantation of embryonic sensory neurons may improve sensory neurons in the skeletal muscle and reinnervate the muscle spindles.

**Materials and methods:**

We collected sensory neurons from dorsal root ganglions of 14‐day‐old rat embryos and prepared a rat model of peripheral nerve injury by performing sciatic nerve transection and allowing for a period of one week before which we performed the cell transplantations. Six months later, the morphological changes of muscle spindles in the cell transplantation group were compared with the naïve control and surgical control groups.

**Results:**

Our results demonstrated that transplantation of embryonic dorsal root ganglion cells induced regeneration of sensory nerve fibre and reinnervation of muscle spindles in the skeletal muscle. Moreover, calbindin D‐28k immunoreactivity in intrafusal muscle fibres was maintained for six months after denervation in the cell transplantation group, whereas it disappeared in the surgical control group.

**Conclusions:**

Cell transplantation therapies could serve as selective targets to modulate mechanosensory function in the skeletal muscle.

## 1 INTRODUCTION

Denervation of skeletal muscle results in extrafusal muscle fibre necrosis, connective tissue hyperplasia and atrophy of muscle spindles.^1^, ^2^ After surgical repair of a cut peripheral nerve, the skeletal muscle is reinnervated and restores force generation. However, it does not regain the ability of stretch reflex and locomotor control, because of the failure to reinnervate muscle spindles or mis‐innervation of other muscle spindles.^3^ Muscle spindles are involved in stretch reflexes and locomotor control.^4^ Loss of sensory feedback from muscle spindles results in disruption of the proprioception of muscle movement.

Recent studies using cultured cells into nerve conduits have offered a novel approach for combining nerve repair and enhanced axonal regeneration.^5^ Schwann cells are essential for peripheral nerve regeneration, and transplanted Schwann cells have been shown to enhance axonal regeneration.^6^ Although there have been a few reports^2^, ^7^, ^8^, ^9^ investigating morphological changes and functional recovery of muscle spindles after preservation or repair of afferent innervation, to the best of our knowledge, there have been no reports that examined reinnervation of muscle spindles using cell transplantation procedures. Thus, we transplanted embryonic dorsal root ganglion (DRG) cells, including Schwann cells and sensory neurons, into a Wallerian‐degenerating nerve and evaluated muscle spindles, the proprioceptive receptors in the skeletal muscles. The purpose of this study was to investigate whether transplantation of DRG neurons into the peripheral nerve improves reinnervation of muscle spindles in the rat.

## 2 MATERIALS AND METHODS

### 2.1 Animal models

All animal maintenance procedures and experimental protocols used in this study were approved by the Animal Ethics Research Committee at Nagoya University. The recipients were 8‐week‐old male Fischer 344 rats (Japan SLC), which were assigned to three groups: naïve control (n = 12), surgical control (n = 12) and cell transplantation (n = 12). All surgical procedures were performed under a surgical microscope and 2% isoflurane anaesthesia. The naïve control group did not receive nerve transection or cell transplantation. In the surgical control and cell transplantation group, the sciatic nerve was transected at the mid‐thigh. The proximal stump was ligated using 4‐0 nylon and was sutured into the gluteus muscle to prevent natural regeneration. The distal stump of a sciatic nerve was divided into the tibial and peroneal nerves, which were ligated using 5‐0 nylon.

### 2.2 Cell preparation and transplantation

**Figure 1:**
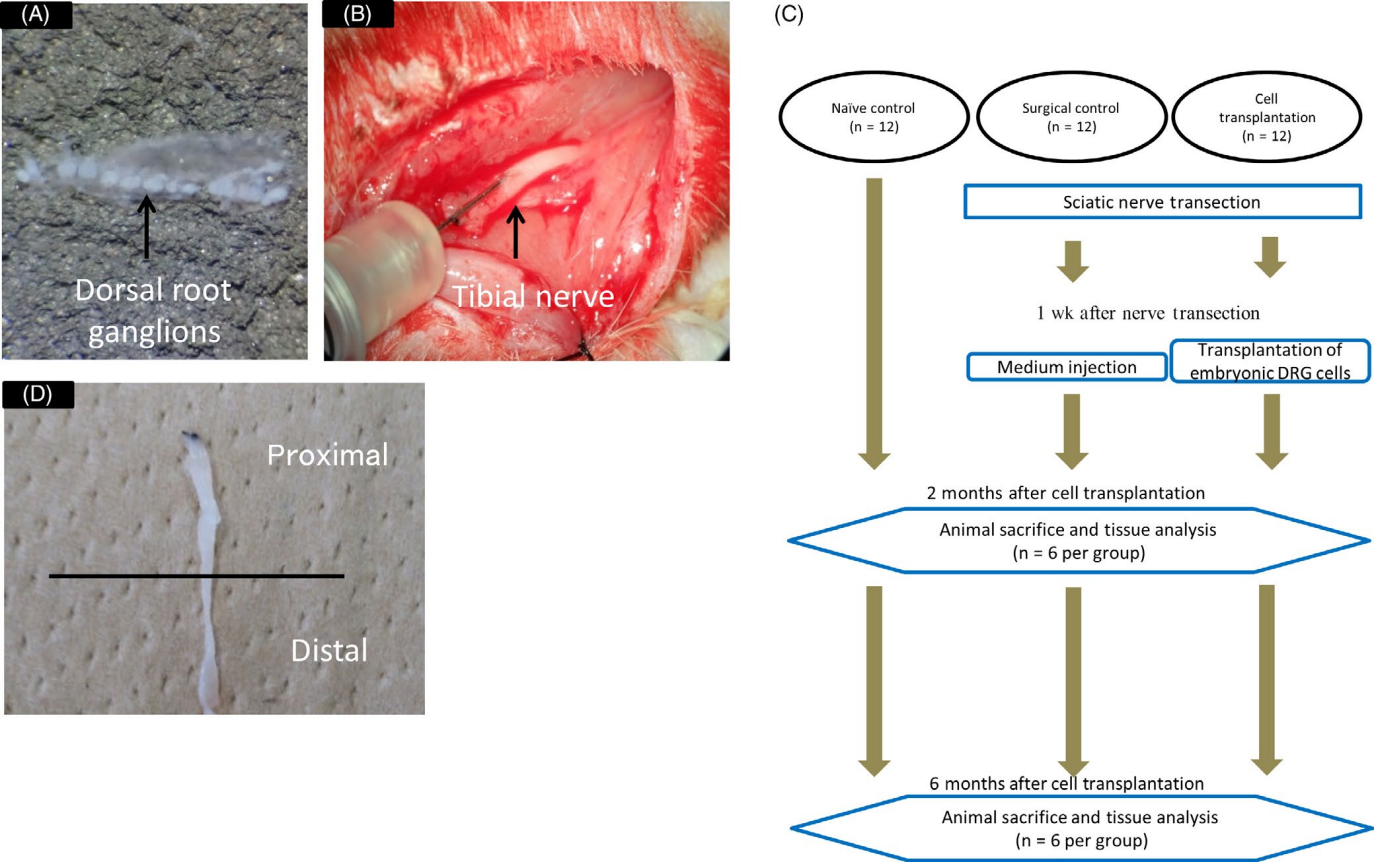
Cell preparation and transplantation. (A) Image showing the dissected DRGs obtained from a 14‐day rat embryo. (B) Image showing DRG cells injection into the distal stumps of the tibial nerve. (C) Flow chart depicting experimental steps. (D) The harvested tibial nerve that was separated into proximal and distal halves at two and six months after cell transplantation

One week after nerve transection, embryonic DRG cells were prepared for injection into the distal stump of the tibial nerve. Three female Fischer 344 rats were used to obtain DRG cells from their 14‐day‐old embryos. The pregnant rats were anesthetized, and their embryos were removed from the uterus. DRGs were resected under a surgical microscope (Figure 1A) and placed into Hanks’ balanced salt solution (Life Technologies Japan). Embryonic DRG cells were dissociated using a papain‐containing dissociation solution (MB‐X0801, Sumitomo Bakelite Co.) and suspended in culture medium (MB‐X9501, Sumitomo Bakelite Co.). Recipient rats were anesthetized, and one million embryonic DRG cells in 10 μL culture medium were slowly injected into the distal stumps of the tibial nerve, using a Hamilton syringe with a 30‐G needle; medium without any cells was injected into the surgical control side (Figure 1B). The injection point was about 20 mm proximal to the entry into the gastrocnemius and soleus muscles.

### 2.3 Animal sacrifice

All rats were sacrificed under anaesthesia and perfused with 50 mL 0.9% saline, followed by 200 mL 4% paraformaldehyde in 0.1 mol/L phosphate buffer (pH 7.4). For chronological analysis, the entire tibial nerves and soleus muscles were frozen at 2 and 6 months after cell transplantation (Figure 1C).

### 2.4 Tibial nerve analysis

The harvested 20 mm of the tibial nerve was separated into proximal and distal halves (Figure 1D). The tissues were cryoprotected in 30% sucrose and then frozen in dry ice‐cooled isopentane. The proximal half of the tibial nerve was used to investigate the properties of cells in the transplantation site. Longitudinal sections of 30 µm were cut and stained with Hoechst (1:1000; Dojindo, Kumamoto) and neural 3‐colour immunocytochemistry kit (1:10; R&D Systems), which includes anti‐glial fibrillary acidic protein, anti‐oligodendrocyte marker O4 and anti‐β3‐tubulin antibodies. The distal half of the tibial nerve was used for morphometric analysis of nerve fibre density. Axial frozen sections of the nerve (30 µm) were cut and stained with FluoroPan neuronal marker Alexa Fluor 488 conjugate (1:200; Millipore) including four antibodies (anti‐neuronal nuclei, anti‐microtubule‐associated protein 2, anti‐β3‐tubulin and anti‐neurofilament‐H antibodies). Three arbitrary points were automatically selected under an all‐in‐one type fluorescence microscope BZ‐9000 (Keyence), and the mean nerve fibre density was calculated using imagej software (National Institutes of Health) and statistically analysed.

### 2.5 Neuromuscular junctions and muscle spindles (intrafusal muscle fibres and capsules)

The triceps surae muscle was dissected from its origin and insertion. Then, it was divided into the soleus and gastrocnemius muscles. The soleus muscle was weighed and cut into 100‐µm‐thick cross sections. The weight of the soleus muscle expressed as a percentage of body weight. Acetylcholine receptors (AChR) were identified by immunoreactivity to Alexa Fluor 594‐conjugated α‐bungarotoxin (1:300; Molecular Probes) and assessed morphologically. FluoroPan neuronal marker was used as a nerve fibre marker (1:200). Muscle spindles and capsules were identified by immunoreactivity to calbindin D‐28k (1:1000; Millipore). Muscle spindles were quantified by counting the number of structures per soleus muscle. BZ‐9000 microscope and A1Rsi confocal fluorescence microscopy (Nikon) were used to assess neuromuscular junction synapse‐like structures and muscle spindles with sensory terminals.

### 2.6 Statistical analysis

Statistical analysis was performed using Ekuseru‐Toukei 2010 software (Social Survey Research Information Co., Ltd). All data are reported as mean ± standard error of the mean. We performed statistical analysis with one‐way ANOVA for multiple comparisons to determine the differences in the histological examination. A value of *P* < 0.05 was considered statistically significant for all variables.

## 3 RESULTS

### 3.1 Histological analysis in the transplantation site

**Figure 2:**
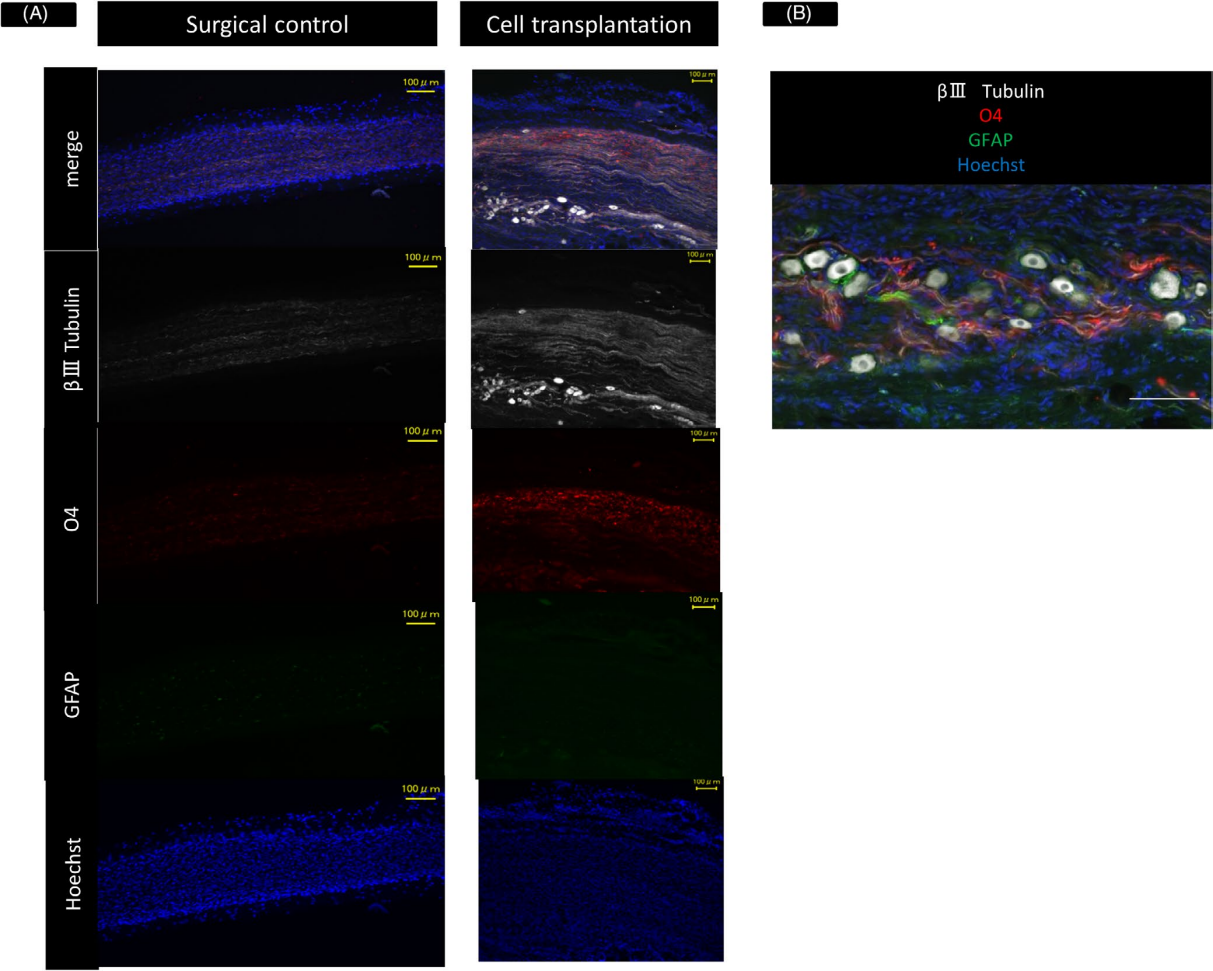
Properties of cells in the transplantation site. (A) Longitudinal sections of the proximal tibial nerve isolated from rats in the surgical control and cell transplantation groups, stained with anti‐β3‐tubulin to mark neurons, anti‐oligodendrocyte marker O4 and Hoechst. White = β3‐tubulin; red = oligodendrocyte marker; blue = Hoechst. Scale bars are 100 μm. (B) Confocal image showing neurons surrounded by GFAP or O4 immunoreactive tissues in the transplantation site. White = β3‐tubulin; red = oligodendrocyte marker; blue = Hoechst; green = glial fibrillary acidic protein. Scale bars are 100 μm

In the cell transplantation group, nerve axons and cell bodies were identified by immunoreactivity to β3‐tubulin. O4‐positive tissue was also found within the transplantation site of the proximal tibial nerve (Figure 2A). In contrast, there were no nerve axons and cell bodies in the surgical control group (Figure 2A). Confocal images depicting neurons surrounded by GFAP and O4 immunoreactive tissues in the transplantation site are shown in Figure 2B.

### 3.2 Nerve fibre analysis

**Figure 3:**
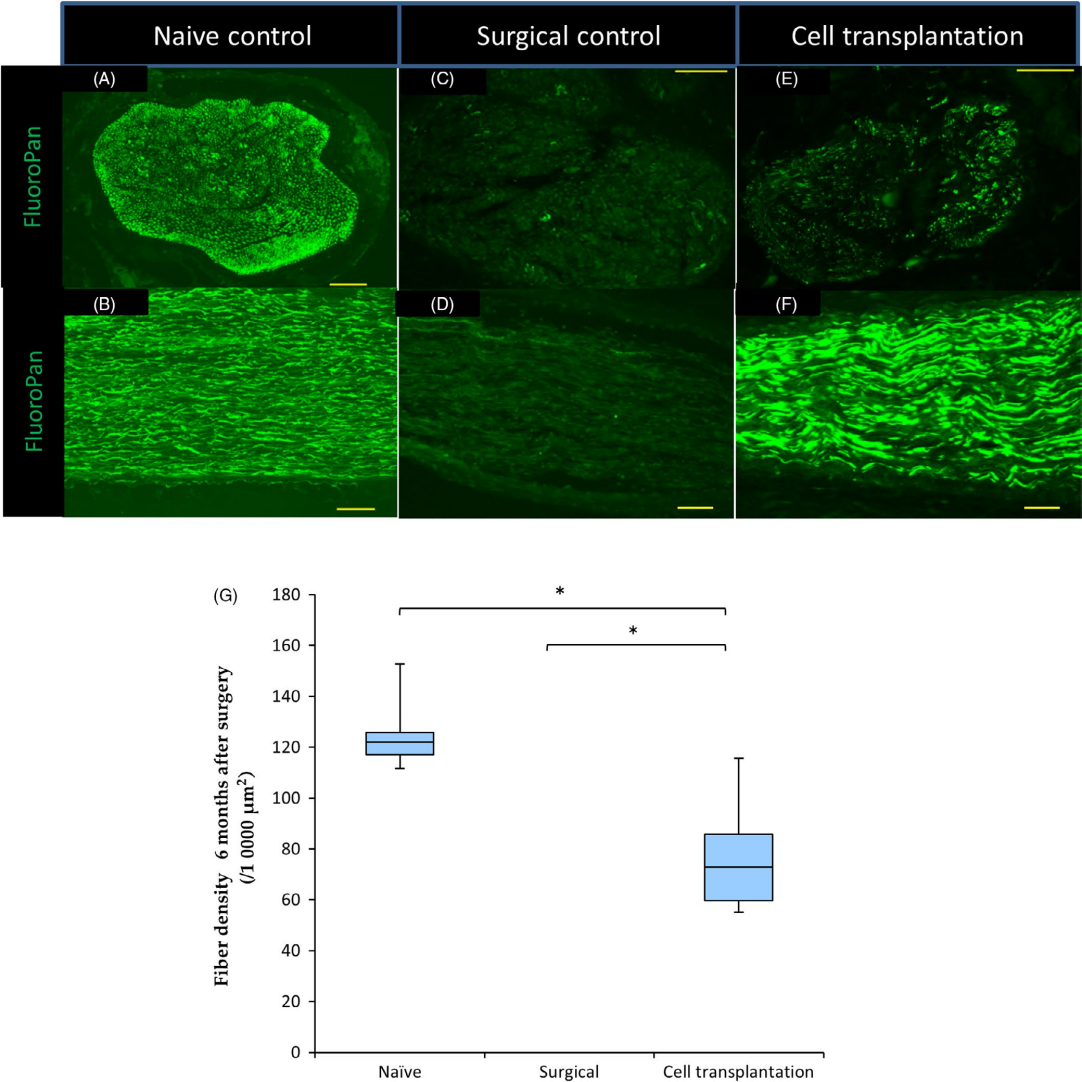
Tibial nerve innervation improves after sensory cell transplantation. (A‐F) Representative axial (A,C,E) and longitudinal sections (B,D,F) of the tibial nerve from naïve control (A,B), surgical control (C,D) and cell transplantation (E,F) rats stained with the FluoroPan neuronal marker. Green = FluoroPan. Scale bars are 100 μm. (G) Quantification of fibre density in the tibial nerve six months after cell transplantation ((n = 6 per group) *P < 0.001). The average nerve fibre density in the cell transplantation group was lower than that in the naïve control (*P* = 0.0003), but was higher than that in the surgical control group (*P* = 0.000006)

Six months after cell transplantation, there were many nerve fibres stained with FluoroPan neuronal marker in the naïve control group (Figure 3A,3B), whereas we did not find any myelinated axons in axial and longitudinal frozen sections of the distal tibial nerve in the surgical control group (Figure 3C,3D). In contrast, in the cell transplantation group, many nerve fibres were observed (Figure 3E,3F). The average nerve fibre density was 125.1 ± 5.9 × 10^−4^ µm^2^ in the naïve control group. The density in the cell transplantation group (77.0 ± 9.4 × 10^−4^ µm^2^) was lower than that in the naïve control, but was improved by approximately 60% compared to the naïve control group.

### 3.3 Neuromuscular junctions

**Figure 4:**
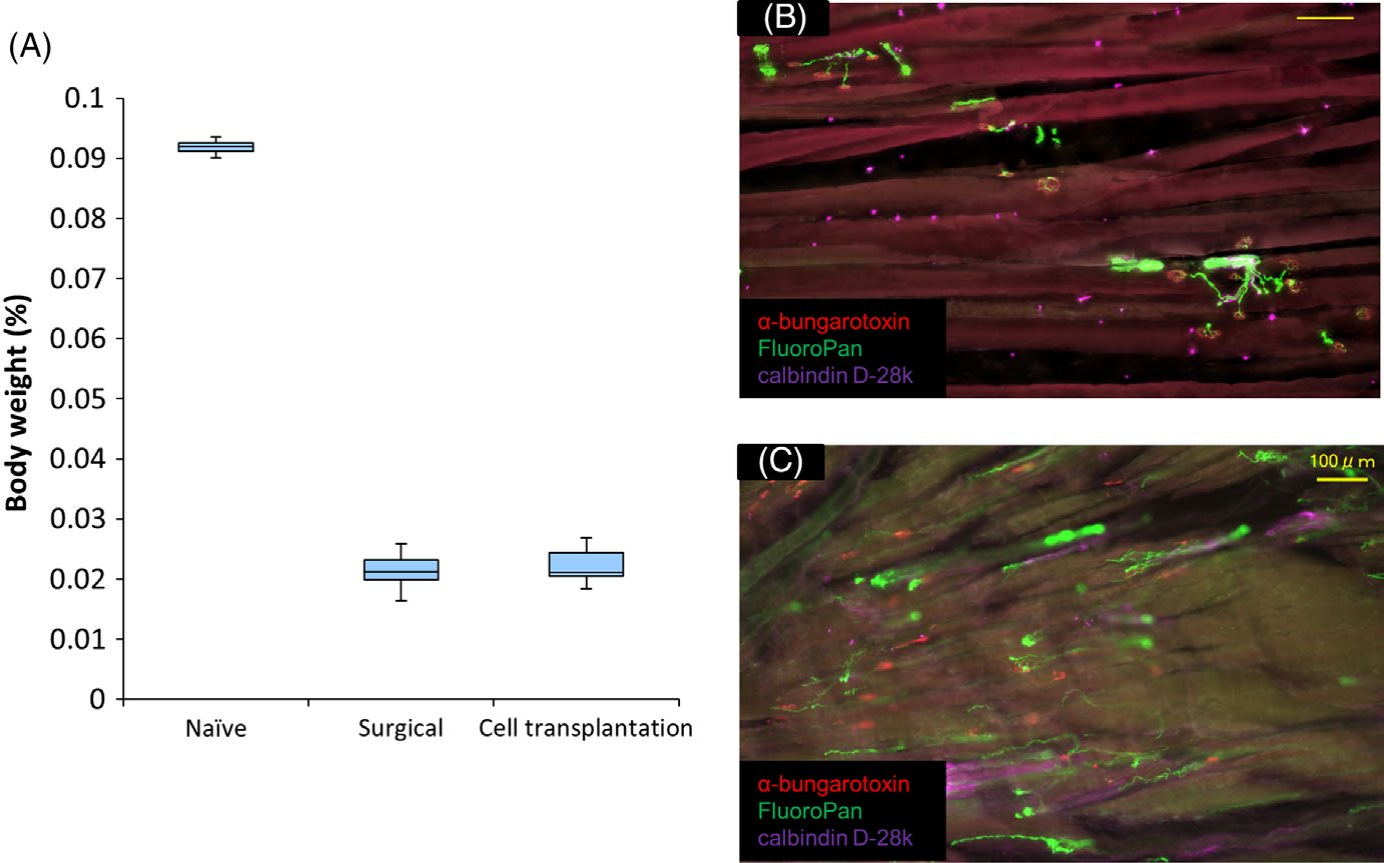
Neuromuscular junctions and the weight of soleus muscle. (A) Quantification of the weight of soleus muscle in the three groups at six months after cell transplantation. (n = 6 per group). There were no significant differences between the weight of soleus muscle in the surgical control and that in the cell transplantation groups (*P* = 0.85). (B,C) Longitudinal sections of the soleus muscle stained with α‐bungarotoxin, calbindin D‐28k and FluoroPan neuronal marker in naïve control (B) and cell transplantation (C) groups. Red = α‐bungarotoxin; green = FluoroPan; purple = calbindin D‐28k. Scale bars are 100 μm

Six months after cell transplantation, the average percentage of body wet weight of the soleus muscle was 0.092 ± 0.001, 0.021 ± 0.001 and 0.022 ± 0.001% in the naïve control, surgical control and in the cell transplantation groups, respectively. There were no significant differences between surgical control and cell transplantation groups at 6 months after cell transplantation (Figure 4A). The neuromuscular junctions were innervated with nerve fibres in the naive control group (Figure 4B). In the cell transplantation group, nerve fibres reached into the muscle, but no neuromuscular junctions at the site of AChR clusterings were reinnervated (Figure 4C).

### 3.4 Muscle spindles

**Figure 5:**
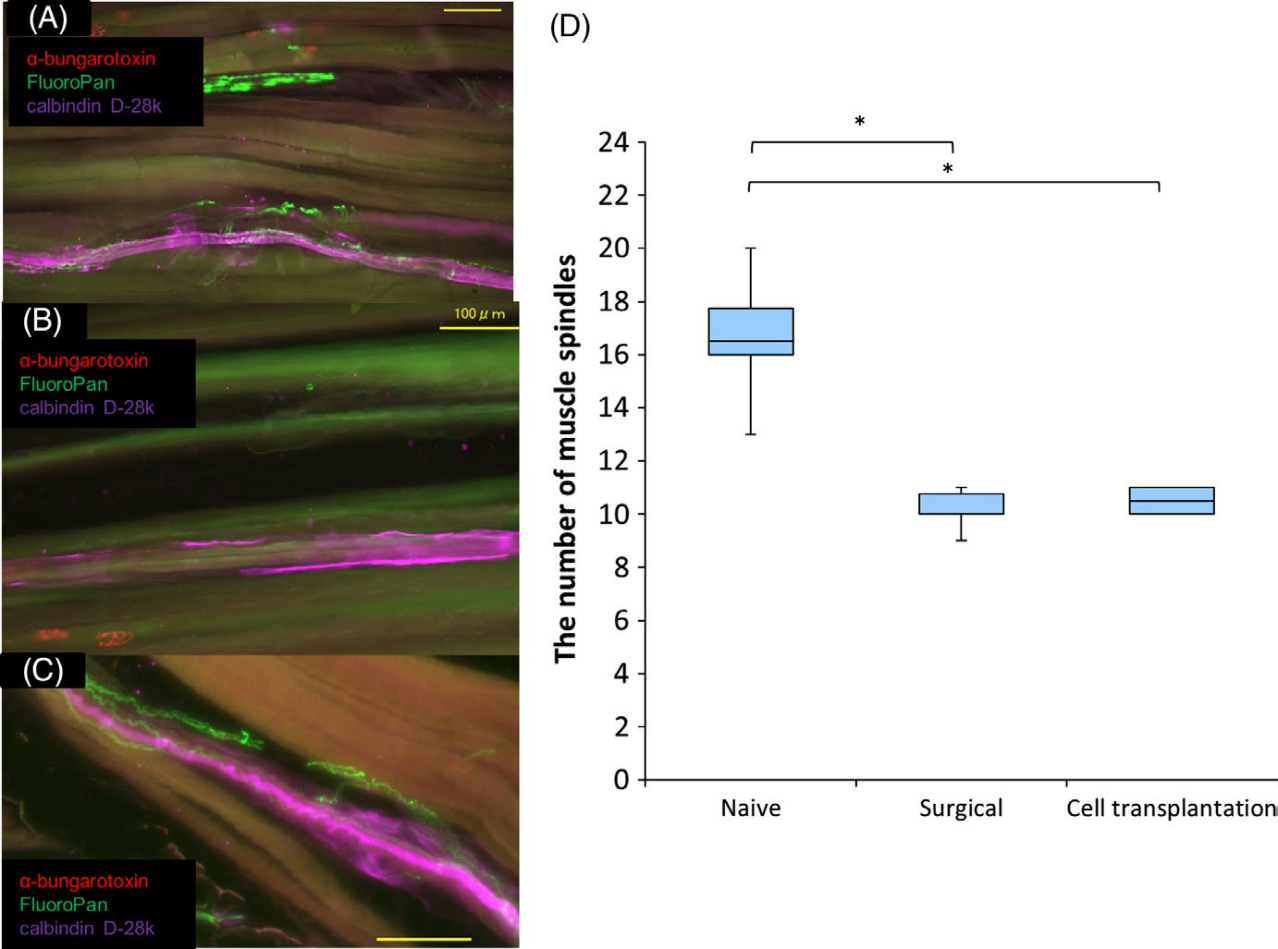
Number of muscle spindles at two months after cell transplantation. (A‐C) Longitudinal sections of the soleus muscle stained with α‐bungarotoxin, calbindin D‐28k, and FluoroPan neuronal marker in naïve control (A), surgical control (B) and cell transplantation (C) groups. Red = α‐bungarotoxin; green = FluoroPan; purple = calbindin D‐28k. Scale bars are 100 μm. (D) Quantification of the number of muscle spindles in the three groups at two months after cell transplantation ((n = 6 per group) *P < 0.001). The average number of muscle spindles was lower in the surgical control (*P* = 0.000005) and cell transplantation groups (*P* = 0.000008) than in the naïve control group

Two months after cell transplantation, all muscle spindles were innervated with nerve fibres in the naïve control group (Figure 5A). Almost half of muscle spindles were reinnervated with nerve fibres in the cell transplantation but not in the surgical control group (Figure 5B,5C). The average number of muscle spindles was lower in the surgical control (10.2 ± 0.31) and cell transplantation groups (10.5 ± 0.22) than in the naïve control group (16.7 ± 0.96; Figure 5D).

**Figure 6:**
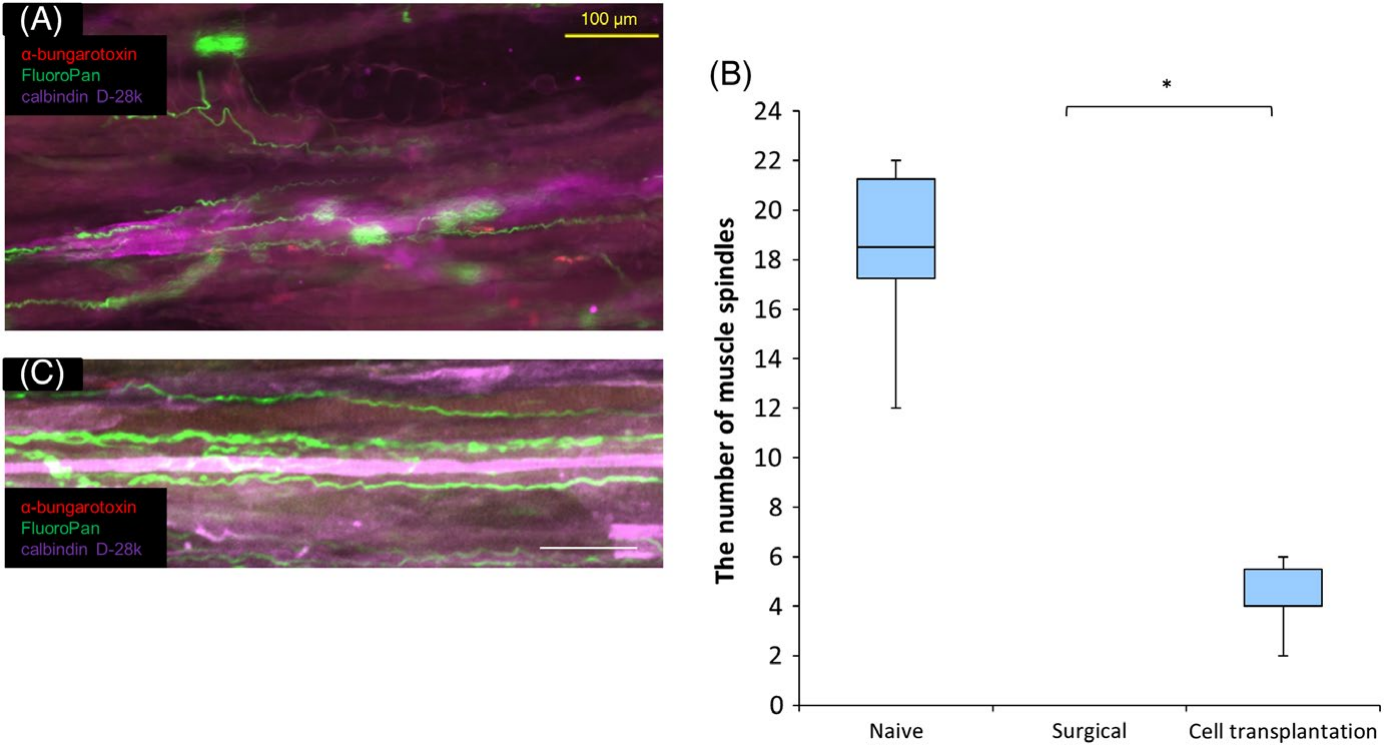
Number of muscle spindles at six months after cell transplantation. (A) Longitudinal sections of the soleus muscle stained with α‐bungarotoxin, calbindin D‐28k and FluoroPan neuronal marker in the cell transplantation group. Red = α‐bungarotoxin; green = FluoroPan; purple = calbindin D‐28k. Scale bar is 100 μm. (B) Confocal image showing muscle spindles reinnervated with nerve terminals. Red = α‐bungarotoxin; green = FluoroPan; purple = calbindin D‐28k. Scale bar is 100 μm. (C) Quantification of the number of muscle spindles in the three groups at six months after cell transplantation ((n = 6 per group) *P < 0.05). The average number of muscle spindles was significantly higher in the cell transplantation (P = 0.014) than in the surgical control group

Six months after cell transplantation, the average number of muscle spindles was 18.3 ± 1.52 in the naïve control group. There were no muscle spindles in the surgical control group. In contrast, in the cell transplantation group, some muscle spindles were reinnervated with nerve fibres, and their calbindin D‐28k immunoreactivity in intrafusal muscle fibres was maintained for six months after denervation (Figure 6A). The average number of muscle spindles was significantly higher in the cell transplantation (4.33 ± 0.62, *P* < 0.05) than in the surgical control group (Figure 6C). Regenerated nerves showed reinnervation of muscle spindles in the skeletal muscle in the cell transplantation group. While most of the muscle spindles exhibited non‐spiral and irregular morphology, some showed a spiral appearance (Figure 6B).

## 4 DISCUSSION

The purpose of this study was to investigate whether transplantation of DRG neurons into peripheral nerves improve reinnervation of muscle spindles in the rat. Our results demonstrated that embryonic DRG cell transplantation improved sensory nerve fibres and reinnervation of muscle spindles in the skeletal muscle. Moreover, calbindin D‐28k immunoreactivity in intrafusal muscle fibres was maintained six months after denervation in the cell transplantation group.

Muscle spindles are proprioceptive receptors in skeletal muscle that respond to the length and tension of the muscle.^10^ It has been suggested that muscle spindles play an important role in the maintenance of postural stability and balance during locomotion^11^, ^12^ and that structural changes occur in them following peripheral nerve injury.^3^ Almost all muscle spindles atrophy and finally disappear after denervation.^13^ However, the number and morphologic deterioration of muscle spindles improve when the muscle is reinnervated.^7^ Regeneration of muscle spindles is dependent upon survival of spindle capsules and intrafusal muscle fibres.^14^ Muscle spindles are innervated with γ‐motorneurons and Ia and II sensory neurons. It is suggested that γ‐motorneurons affect the maintenance of muscle spindle formation. However, motorneuron innervation is not essential for the formation and differentiation of muscle spindles in reinnervated muscles of neonatal rats.^15^ In the present study, the mere transplantation of embryonic sensory neurons could maintain muscle spindle formation, as well as the preservation of spindle capsules and regeneration of intrafusal muscle fibres after denervation. These results reveal that sensory nerve reinnervation is important to preserve the muscle spindles in regenerating mammalian skeletal muscle.

Regarding the morphological changes of muscle spindles, some authors have reported the influence of peripheral nerve injury, hindlimb unloading and age.^3^, ^8^, ^16^, ^17^ After nerve repair, sensory terminals of regenerated muscle spindles are tapered or have irregular forms, but the stretch reflex gradually recovers to normal levels.^18^, ^19^, ^20^ Age causes remarkable changes in the structures, not only in the intrafusal muscle but also in sensory nerve endings in muscle spindles.^21^ Sensory terminals of old rats appear somewhat irregular and less crowded. Primary endings of aged rat muscle spindles are less spiral or non‐spiral in appearance.^22^ Irregular innervation was also found in the muscle spindles of diabetic mice after Ia axonal degeneration and regeneration.^23^ In the present study, we found that most muscle spindles in the rat are non‐spiral and irregular in appearance after cell transplantation. This morphology may affect muscle spindle function, since sensory nerve terminals are important to mechanosensory function.

Calbindin D‐28k is a vitamin D‐dependent calcium‐binding protein.^24^ Calcium binding proteins buffer calcium, thus preserving intracellular Ca^2+^ homoeostasis.^25^ Calbindin D‐28k has been shown to have functions as Ca^2+^ buffer, transporter and sensor.^26^ (Schmidt H, 2012). Calbindin D‐28k immunoreactivity has been demonstrated in central and peripheral neurons^25^, ^27^ and was also detected in the kidney and pancreas.^28^ Furthermore, calbindin D‐28k is found in sensory pathways such as cones and horizontal cells in the retina, cochlear and vestibular hair cells in the inner ear, intrafusal muscle fibres, the spindle capsule and the perineural sheath of nerves.^29^, ^30^, ^31^ Calbindin D‐28k is involved in adjustment of intracellular calcium levels and mediation of Ca^2+^ dependent events, when intrafusal muscle fibres contract. In a rat denervation model, it was shown that calbindin D‐28k immunoreactivity of intrafusal muscle fibres remains, although reduced, for 1 month. After two months of denervation, calbindin D‐28k immunoreactivity is not seen in intrafusal muscle fibres, although it remains in the spindle capsule.^31^ In the present study, we revealed that some muscle spindles were reinnervated with sensory nerve fibres and that intrafusal muscle fibres maintained calbindin D‐28k immunoreactivity for six months in the sensory cell transplantation group. It is suggested that Ca^2+^ homeostasis in the intrafusal muscle fibres is influenced by a trophic effect produced by the regenerated sensory nerves fibres. In Ia peripheral endings, synaptic vesicles contain a lot of glutamate and undergo recycling in a Ca^2+^‐modulated manner to enhance terminal excitability during muscle stretch.^32^

In our previous report, transplantation of neural stem cells into the peripheral nerve revealed a structure resembling spinal cord tissue, and all types of central nervous system cells, such as neurons, astrocytes and oligodendrocytes, were identified, by electron microscopy analysis.^33^ In the present study, we could also find neurons surrounded by GFAP and O4 immunoreactive tissues in the transplantation site. While there is a possibility that support by central nervous system cells is indispensable, transplantation of DRG neurons improved sensory nerve fibres and maintained muscle spindle formation. Preservation of afferent innervation prior to motor nerve repair demonstrated functional recovery of skeletal muscles after prolonged denervation.^2^ Therefore, transplantation of DRG neurons may result in functional recovery of long‐term denervated muscles when combined with motor nerve repair. In addition, preservation of afferent innervation has been reported to reduce contractures after neonatal brachial plexus injury, which is associated with muscle spindle degeneration.^9^ In this study, transplantation of DRG neurons showed morphological preservation of muscle spindle structures and may have an effect on treatment of contractures after neonatal brachial plexus injury.

A limitation of this study is the lack of functional analysis of muscle spindles in the skeletal muscle. To more clearly investigate the effect of cell transplantation, the rat tibial nerve was cut off from the central nerve in the current animal model. Because the nerve amputation model entailed a separation from the central nerve, it was difficult to perform functional tests on the tibial nerves. In order to confirm a behavioural and functional evaluation, future studies should assess another tibial nerve injury model that maintains the central nerve.

In conclusion, the present study demonstrated that transplantation of embryonic sensory neurons improved sensory nerve fibres and reinnervation of muscle spindles in the skeletal muscle. Moreover, immunoreactivity of calbindin D‐28k in intrafusal muscle fibres was maintained for six months after denervation in the cell transplantation group. In the future, cell transplantation therapies could serve as selective targets to modulate mechanosensory function in the skeletal muscle.

## CONFLICT OF INTEREST

The authors have declared that there is no conflict of interest.

## AUTHOR CONTRIBUTIONS

KA designed the study, executed the experiments, interpreted the data and drafted the manuscript. TN executed the experiments and interpreted the data. KT, HI, TN, KI and TO executed the experiments. SK executed the experiments and performed the literature review. MY and MT interpreted the data. HH conceived of and supervised the study, interpreted the data and reviewed the manuscript. All authors approved the final version of the manuscript.

## Data Availability

The data that support the findings of this study are openly available in “figshare” at https://doi.org/10.6084/m9.figshare.7856738.v1
